# Sex differences in functional and structural alterations of hippocampus region in chronic pain: a DTI and resting-state fMRI study

**DOI:** 10.3389/fnins.2024.1428666

**Published:** 2024-09-06

**Authors:** Jun-Zhi Zhou, Jie Deng, De-Xing Luo, Jing-Wen Mai, Jia-Yan Wu, Yu-Juan Duan, Bo Dong, Wen-Jun Xin, Ting Xu, Jia-You Wei

**Affiliations:** ^1^Center for Infection and Immunity and Guangdong Provincial Engineering Research Center of Molecular Imaging, The Fifth Affiliated Hospital of Sun Yat-sen University, Zhuhai, Guangdong, China; ^2^Neuroscience Program, Zhongshan School of Medicine, Guangdong Province Key Laboratory of Brain Function and Disease, Department of Physiology and Pain Research Center, Sun Yat-sen University, Guangzhou, China; ^3^Department of Anesthesiology, The First Affiliated Hospital, Sun Yat-sen University, Guangzhou, China; ^4^Department of Anesthesiology, Huizhou Central People’s Hospital, Huizhou, China; ^5^Guangdong-Hong Kong-Macao University Joint Laboratory of Interventional Medicine, The Fifth Affiliated Hospital, Sun Yat-sen University, Zhuhai, China; ^6^Guangdong-Hong Kong-Macao Greater Bay Area Center for Brain Science and Brain-Inspired Intelligence, Zhuhai, China

**Keywords:** resting-state functional magnetic resonance imaging, diffusion tensor imaging, chronic pain, sex difference, hippocampus region

## Abstract

**Introduction:**

It is well known that there are significant differences in the prevalence of chronic pain between males and females. Human and animal imaging studies have shown that chronic pain profoundly alters the structure and function of brain regions. However, there is limited research on the sex-specific mechanisms underlying the brain plasticity and adaptive changes associated with chronic pain. In this article, we conducted a multimodal study to evaluate how nerve injury-induced chronic pain affects the brain.

**Methods:**

Male and female Sprague-Dawley (SD) rats with spared nerve injury (SNI) model underwent resting-state functional magnetic resonance imaging (rs-fMRI) (male sham group: *n* = 18; male SNI group: *n* = 18; female sham group: *n* = 20; female SNI group: *n* = 18) and magnetic resonance diffusion tensor imaging (DTI) (male sham group: *n* = 23; male SNI group: *n* = 21; female sham group: *n* = 20; female SNI group: *n* = 21) scanning. ICA method, Fractional amplitude of low-frequency fluctuations (fALFF), immunofluorescence staining, and graph theory analysis was utilized to extract the rs-fMRI changes of brain regions of each group.

**Results:**

Using SNI model, which promotes long-lasting mechanical allodynia, we found that neuropathic pain deeply modified the intrinsic organization of the brain functional network in male and female rats (main effect of operation: *F* = 298.449, *P* < 0.001). 64 independent components (ICs) in the brain were divided and assigned to 16 systems. In male rats, we observed significant alterations in the microstructure of the hippocampal cornu ammonis 1 and cornu ammonis 2 (CA1/CA2) region, as indicated by increased mean diffusivity (MD) (CA1_L: *P* = 0.02; CA1_R: *P* = 0.031; CA2_L: *P* = 0.035; CA2_R: *P* = 0.015) and radial diffusivity (RD) (CA1_L: *P* = 0.028; CA1_R: *P* = 0.033; CA2_L: *P* = 0.037; CA2_R: *P* = 0.038) values, along with enhanced activating transcription factor 3 (ATF3) expression. Conversely, in female rats, we found significant increases in the fractional amplitude of low frequency fluctuations (fALFF) value within the hippocampal dentate gyrus (DG) (*F* = 5.419, *P* = 0.023), accompanied by elevated c-Fos signal (*F* = 6.269, *P* = 0.031). Furthermore, graph theory analysis revealed notable differences in the small-world network of the hippocampal system in female rats, characterized by reduced small-world attributes and increased inter-nodal transmission efficiency.

**Discussion:**

Our study indicates sex differences in structural and functional alterations in the hippocampal system in rats under chronic pain conditions. The results suggest that the hippocampus system plays an important role in the different mechanisms of chronic pain in different sexes. These findings provide reliable insights to explore the complex mechanisms underlying sex differences in chronic pain.

## 1 Introduction

Chronic pain affects over 20% of adults and is one of the leading causes of disability worldwide (Goldberg and McGee, 2011). Women are particularly affected by chronic pain. Approximately half of the chronic pain subtypes are more common in women, with only 20% higher prevalence rate in men. Previous preclinical pain research has predominantly focused on male animals. However, recent studies involving both sexes have increasingly revealed significant sex differences in the physiological and pathological mechanisms of pain. These differences include the involvement of different genes and proteins in pain processes in different sexes, as well as clear interactions between hormones and the immune system that influence pain signal transmission (Mogil and Chanda, 2005; Mogil, 2020; Mogil, 2012).

Neuropathic pain, caused by conditions such as nerve injury, is a common form of chronic pain and a clinical challenge that affects many patients. Clinical and preclinical research has found that sexual dimorphism also exists in this context, referring to the differences between males and females in terms of neuropathic pain (Mogil, 2020; Bartley and Fillingim, 2013). Although the role of sex differences in the mechanisms of neuropathic pain has garnered attention in the academic community, there is still a relative lack of research on the brain plasticity (structural and functional changes) of male and female rats following neurotrauma-induced chronic pain.

Nowadays, neuroimaging techniques are widely used to study the role of the central nervous system in chronic pain maintenance and development in pain neuroscience (Martucci and Mackey, 2018). Functional magnetic resonance imaging (fMRI) is a technique that analyzes brain activity by capitalizing on the blood oxygen level dependent (BOLD) phenomenon. When the local deoxyhemoglobin level decreases, it leads to an increase in the T2-weighted signal on fMRI images, indicating an increase in oxygen consumption and enhanced neuronal activity in that particular brain region (Glover, 2011). Building upon this, resting state functional magnetic resonance imaging (rs-fMRI) provides a non-invasive, repeatable, and high spatial and temporal resolution approach to observe the underlying brain function in pathological conditions of the central nervous system. This technique offers greater possibilities for deciphering the mechanisms and potential treatments of diseases such as chronic pain, Alzheimer’s disease, and post-traumatic stress disorder (Davis and Moayedi, 2013; Dopfel et al., 2019; Raimondo et al., 2021). Independent component analysis (ICA) is a commonly used method applied in the analysis of rs-fMRI data in pain neuroscience (Flodin et al., 2014; Napadow et al., 2010). Based on blind source separation algorithm, ICA can decompose the rs-fMRI signals of whole brain voxels into spatially and temporally independent components (ICs), revealing functional connectivity and network characteristics between different brain regions (Chakraborty et al., 2020). In addition, some graph theoretical approaches have been utilized to quantitatively map the topological organization of large-scale complex neural systems across the specific regions of interest in the brain (Bullmore and Sporns, 2009). Fractional amplitude of low frequency fluctuations (fALFF) is reliable scan indicator in rs-fMRI to reflect the regional spontaneous neural activity and metabolic demand of brain regions (Huang et al., 2022; Lin et al., 2022). Structurally, magnetic resonance diffusion tensor imaging (DTI) is a special types of magnetic resonance imaging technology that uses the diffusion characteristics of water molecules in tissues to generate images. Currently, DTI is an important technology for studying changes in structure of white matter in brain in health and disease (Soares et al., 2013). In contemporary neuroscience research, there is a growing trend towards integrating microstructural alterations assessed through DTI with investigations of brain functionality using rs-fMRI (Le Bihan, 2012).

To deepen our understanding of the possible neurobiological mechanisms of sex differences in chronic pain, we performed rs-fMRI and DTI signals to compare the sex differences of rats’ brain in neuropathic pain models. Specifically, independent components (ICs) were extracted. Based on the main regions of each IC, we separated them into several systems according to *The Rat Brain in Stereotaxic Coordinates-Fifth Edition-Elsevier* (Paxinos and Watson, 2004) and previous study of brain networks in rats (Jonckers et al., 2011). Through a progressive integration approach, we attempt to comprehensively explore the accurate and precise changes in the functionality and activity of brain regions associated with neuropathic pain induced by nerve injury, considering the sex-specific differences.

## 2 Methods and materials

### 2.1 Animals

Male and female Sprague-Dawley (SD) rats (7∼8 weeks old, 200∼250 g) were obtained from the Institute of Experimental Animals of Sun Yat-sen University. Rats were housed in cages (4 rats per cage) with controlled ambient temperature (22∼24°C) and humidity (40∼60%) on a 12/12 h day-dark schedule with food and water ad libitum. All experimental procedures were approved by the Local Animal Care Committee and were performed in accordance with the guidelines of the National Institutes of Health on animal care and the ethical guidelines. Male and female rats were randomly divided into different groups. We made every possible effort to alleviate suffering of animals.

### 2.2 Behavioral test and spared nerve injury (SNI)

**FIGURE 1:**
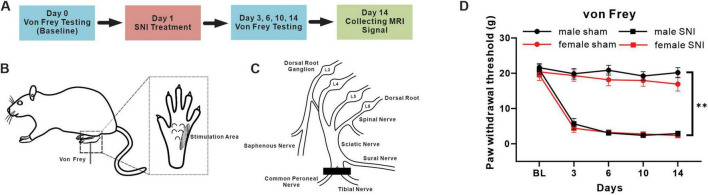
Mechanical pain hypersensitivity induced by SNI in male and female rats. **(A)** Timeline with schematic of experimental design and experiment process. **(B)** A diagram of mechanical pain behavior test and the test range of plantar surface. **(C)** A schematic illustration of the terminal branches of the sciatic nerve and saphenous nerve and SNI incision position after surgical ligation. **(D)** Mechanical paw withdrawal threshold of sham and SNI rats in both sexes (male sham-operation group: *n* = 23; male SNI group: *n* = 21; female sham-operation group: *n* = 20; female SNI group: *n* = 21; **p < 0.01 in main effect of operation).

To investigate mechanical allodynia, each animal was allowed to adaption to a plastic box for 3 consecutive days (15 min/day) before testing. When detecting, the rats were placed in a plastic box on a metal mesh, and von Frey filaments were applied to the lateral aspect of the plantar surface of their left hind paws (Figure 1B). A rapid paw withdrawal or flinching in response to the von Frey filament stimulation of different strength was considered a nociceptive response. The 50% paw withdrawal threshold was determined using a well-established up-down method (Chaplan et al., 1994).

The SNI treatment was performed following the procedure described previously (Decosterd and Woolf, 2000). Briefly, the left tibial and common peroneal branches of the sciatic nerve of rat were cut 2 mm distal to the ligation while under 4% isoflurane anesthesia (Figure 1C). In rats that underwent a sham operation, the left tibial and common peroneal branches of the sciatic nerve were exposed without ligation using the same method.

All rats underwent baseline von Frey test on day 0. The SNI treatment and sham-operation were performed one day after the baseline test. Then von Frey tests were performed on days 3, 6, 10, 14. MRI signals were collecting after behavior tests on days 14 (Figure 1A).

### 2.3 Diffusion tensor imaging (DTI) and resting-state functional magnetic resonance imaging (rs-fMRI) acquisition

MRI data was collected on a 9.4T animal MRI scanner (Bruker Biospin GmbH, Germany). The image signals were collected in the rats with respiratory rate monitored and maintained at 70 times/min after inhalation anesthesia with isoflurane.

During imaging scan, anatomical images were first acquired using a rapid acquisition with relaxation enhancement (RARE) sequence (repetition time (TR) = 5081.564 ms, echo time (TE) = 21.59 ms, matrix size = 150 × 105, field of view (FOV) = 3.0 × 2.1 cm, slice number = 70, slice thickness = 0.4 mm, slice gap = 0, and resolution = 0.20 × 0.20 × 0.40 mm). fMRI data were obtained using a 2D multi-slice, single-shot, gradient-echo EPI sequence, for BOLD the parameters is: TR = 2000 ms, TE = 10.332 ms; flip angle = 90°, matrix size = 100 × 70, FOV = 3.0 × 2.1 cm, slice number = 45, slice thickness = 0.6 mm, slice gap = 0, and resolution = 0.30 × 0.30 × 0.60 mm. For DTI the parameters are: TR = 2557.136 ms, TE = 16.915 ms, flip angle = 90°, matrix size = 100 × 70, FOV = 3.0 × 2.1 cm, slice number = 90, slice thickness = 0.3 mm, slice gap = 0, number of segments = 6, symmetric diffusion gradients were applied with b = 1000 s/mm^2^ in 30 non-collinear directions and b = 0 s/mm^2^.

### 2.4 DTI data pre-processing

We conducted the following operations for the DTI analysis: (1) converted the DTI data to Nifti format, (2) preprocessed and performed all analyses using the tools in FSL (FSL 5.0.9),^1^ (3) corrected for simultaneous multiple eddy current using the FSL eddy current tool, (4) calculated the respective eigenvalues of diffusion tensors (λ1, λ2, and λ3) using FSL, and then calculated the fractional anisotropy (FA), mean diffusivity (MD), axial diffusivity (AD), and radial diffusivity (RD) for each sample corresponding to the brain region based on the SIGMA anatomical rat brain atlas (Barrière et al., 2019).

### 2.5 Rs-fMRI data pre-processing

The resting-state fMRI data were analyzed using the Statistical Parametric Mapping software package (SPM12).^2^ To ensure steady-state longitudinal magnetization, the first 5 volumes of each fMRI scan were discarded. The remaining volumes underwent a series of processing steps, including voxel magnification, slice timing correction, realignment, origin correction, and co-registration to echoplanar imaging (EPI) templates. The resliced volumes were then set to a resolution of 3 × 3 × 3 mm and spatially smoothed using a Gaussian kernel with a full width half-maximum (FWHM) of 6 mm. Additionally, linear detrending was applied to the data. Any rs-fMRI volumes with a relative framewise displacement (FD) greater than 0.3 mm were excluded from further analysis.

Fractional amplitude of low-frequency fluctuations (fALFF) values was calculated using SPM12 software. The time series of each voxel was transformed to the frequency domain using Fast Fourier Transform to obtain the power spectrum. The power spectrum was then square-rooted at each frequency, and the mean square root was computed across the 0.01–0.08 Hz band for each voxel. Finally, fALFF in each voxel was normalized by z-score transformation of the global brain within a brain mask to account for voxels throughout the entire brain. Statistical analysis is based on zfALFF for each voxel.

For independent component analysis (ICA), the preprocessed rs-fMRI data were analyzed using group ICA in Group ICA of fMRI Toolbox (GIFT v4.0a).^3^ Based on the estimation using all subjects, it was determined that there would be 64 ICs. Infomax algorithm was used to extract ICs and ICASSO tool was used to perform stability analysis (ICA was repeated 100 times and ICs with average intra-cluster similarity values > 0.8 were selected). Each mask of ICs was extracted via xjView Toolbox^4^ and the main regions of each IC were extracted by mapping the IC masks to the SIGMA anatomical rat brain atlas (Barrière et al., 2019) via DPABI.^5^

For the graph theory analysis, all ICs of the interest system were extracted as the nodes of the network. The network’s edges were determined by considering the partial correlations between the mean time series of each pair of nodes, while excluding the effects of the other nodes and representing their conditional dependences. The Pearson correlation coefficients for all possible pairs of nodes were computed and converted to z-scores using Fisher’s z transformation to improve the normality of the distribution. Each subject’s matrix was transformed into a matrix with absolute values. Next, the matrices were converted to undirected binary matrices based on a predefined threshold, a range of sparse thresholds S (aij = 1, if the absolute correlation between regions i and area j exceeded the threshold, otherwise aij = 0). Based on previous studies and the data from our present experiment, the threshold range generated by this process was 0.27 ∼ 0.5, and the interval was 0.03 (Fornito et al., 2010). Then we calculated both the global and nodal metrics at every sparsity level using Graph theoretical Network Analysis toolbox (GRETNA).^6^ The global network metrics includes parameters of small-worldness (clustering coefficient (Cp), characteristic path length (Lp), normalized clustering coefficient (γ), normalized characteristic path length (λ), and small-worldness (σ). γ = Cp/Cprand, λ = Lp/Lprand, Cprand and Lprand were calculated from a random network and parameters of network efficiency [global network efficiency (Eglob) and local network efficiency (Eloc)]. Furthermore, we choose betweenness centrality (BC) to measure the nodal characteristics of networks.

### 2.6 Immunofluorescence

The rats received anesthesia and were then perfused transcardially with a solution of 0.9% saline, followed by 4% paraformaldehyde (PFA). The brains were fixed in 4% PFA at 4°C for 12 h, and then moved to 20% sucrose for one day, followed by 30% sucrose for three days. Then, brains were sectioned into 25 μm-thick slices. The slices contained brain regions of interest were selected for immunofluorescence staining. Each slice was washed three times in phosphate-buffered saline (PBS), followed by blocking with 0.3% Triton X-100 and 3% (w/v) normal donkey serum for 1 h. Primary antibodies (c-Fos, ab208942, Abcam, USA; ATF3, ab207434, Abcam, USA) were then applied and incubated overnight at 4°C. The slices were rinsed three times in PBS. Next, the slices were incubated with fluorescence-conjugated secondary antibody (Alexa Fluor^®^ 488, 715-545-150, Jackson ImmunoResearch, USA) at room temperature for 2 h. Finally, the slices were coverslipped with antiquenching mounting medium (Beyotime), and examined under a Nikon (Eclipse Ni-E, Nikon, Japan) fluorescence microscope. Images were captured using a Nikon DS-Qi2 camera. The c-Fos^+^ cells density was measured by ImageJ.

### 2.7 Statistical power calculation and sample size

According to the method designed by Desmond and Glover (2002), we assumed that the level of μ_D_ (the difference in means between the experimental and control condition) was 50%, the α_B_ (inter-subject variability) was 0.5% and the α_W_ (intra-subject variability) was 0.75%, at least 12 subjects were required to reach 80% power for the level of alpha at α = 0.05. According to the sample size in many recent fMRI and DTI studies were around 10–20 subjects in each group (Murakami et al., 2023; Pradier et al., 2021; Sanganahalli et al., 2021). The present study, the sample size in DTI analysis is 23 subjects in male sham group, 21 subjects in male SNI group, 20 subjects in female sham group and 21 subjects in female SNI group, respectively. The sample size in fMRI analysis is 18 subjects in male sham group, 18 subjects in male SNI group, 20 subjects in female sham group and 18 subjects in female SNI group, respectively. We try our best to collected as many samples as possible for our study.

### 2.8 Statistical analysis

All results presented as mean ± SEM. The Shapiro-Wilk test was used to check the normality of data. The data of behavioral tests, mean values of DTI metrics (AD, RD, MD, FA values) in each brain region, mean zfALFF values of each ICs, the c-Fos^+^ cells density and area under the curve (AUC) of global and nodal metrics were analyzed by General Linear Model (GLM) with two levels (sex and operation) using IBM SPSS Statistics 26 to test interaction effects, main effects and simple effects. A two-sided *P* < 0.05 was considered statistically significant.

## 3 Results

### 3.1 SNI induced mechanical allodynia in both male and female rats

First of all, we used von Frey fibers to measure the 50% paw withdrawal threshold of rats’ hind paw to examine the effectiveness of the pain model established. The main effect of operation results showed that the mechanical withdrawal threshold significantly decreased and continued until 14 days in rats treated with SNI, which compared with sham group, in both male and female rats (main effect of operation: *F* = 298.449, *P* < 0.001) (Figures 1A–D). The main effect of sex results showed that the mechanical withdrawal threshold was similar between male and female groups (main effect of sex: *F* = 0.046, *P* = 0.832). For males, the mean of paw withdrawal threshold in SNI and sham groups were 7.022 g vs. 20.340 g, which significantly decreased in the male SNI group compared with the male sham group (simple effect of operation in males: *F* = 169.732, *P* < 0.001); while for females, the mean of paw withdrawal threshold in SNI and sham groups were 6.453 g vs. 18.554 g, which significantly decreased in the female SNI group compared with the female sham group (simple effect of operation in females: *F* = 130.749, *P* < 0.001). The above results indicated that the paw withdrawal threshold had significantly decreased both in male and female SNI group. However, no interaction effect between operation and sex was found (sex × operation level interaction: *F* = 0.685, *P* = 0.408), indicating that the impacts of SNI treatment on mechanical allodynia was not significantly different between male and female.

### 3.2 Independent components extracted and systems divided of fMRI data

**FIGURE 2:**
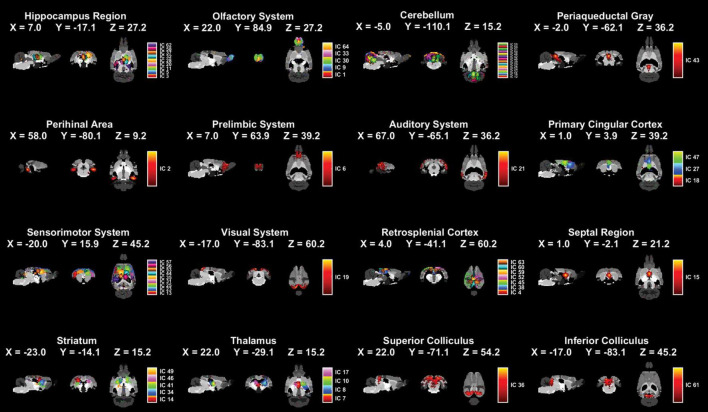
Spatial distributions of 16 brain systems and ICs within each system by applying ICA extraction. The 16 identified systems and core location listed one by one in the figure (male sham group: *n* = 18; male SNI group: *n* = 18; female sham group: *n* = 20; female SNI group: *n* = 18).

The collected fMRI data were separated into independent spatial components using ICA. The computational results show that 64 independent components (ICs) were extracted (Supplementary Figure 1) and these ICs were separated into 16 systems (Figure 2). The names, regional locations, and inclusion of ICs information for the 16 systems were displayed in Figure 2 and Supplementary Table 1.

### 3.3 Sex differences existed in structural changes of hippocampal region of rats in neuropathic pain model

**FIGURE 3:**
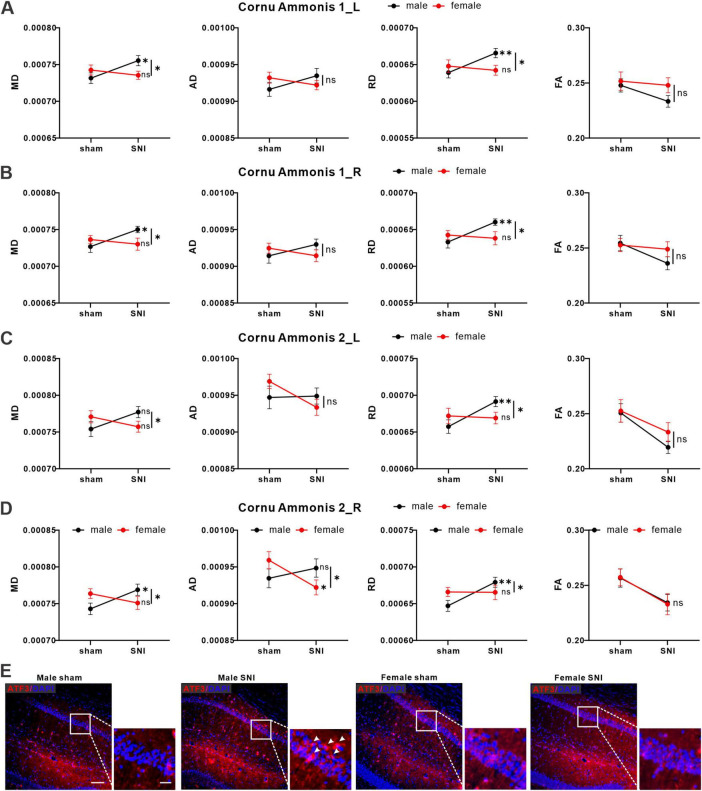
SNI induced changes of DTI signals in hippocampus region of the brain. Average values for MD, AD, RD and FA values in CA1_L **(A)**, CA1_R **(B)**, CA2_L **(C)**, CA2_R **(D)** of male (black icon) and female (red icon) rats (male sham group: *n* = 23; male SNI group: *n* = 21; female sham group: *n* = 20; female SNI group: *n* = 21; * *p* < 0.05, ** *p* < 0.01, ns: no significance). **(E)** Representative immunofluorescence images of ATF3 expression in each group (left bar = 100 μm, right bar = 25 μm).

To investigate potential microstructural changes in brain regions of rats of different sexes caused by nerve injury, we collected and analyzed DTI data. It was found that there were sex differences in the changes of DTI metrics of the bilateral hippocampal cornu ammonis 1 (CA1_L & R) and cornu ammonis 2 (CA2_L & R). The MD, AD, RD and FA values and statistical interaction within CA1_L & R and CA2_L & R were showed in Figure 3. In detail, there were significant interaction effects of MD and RD values between sex and operation in CA1_L & R and CA2_ L & R. In terms of simple effects, MD and RD values were significantly increased in the SNI male group compared with the sham male group in CA1_L & R and CA1_R. For CA2_L, only the RD value was significantly increased in the SNI male group compared with the sham male group. For CA2_R, AD value was significantly decreased in the SNI female group compared with the sham female group (Figures 3A–D). All of the specific statistical parameters were shown in Supplementary Table 2. To further confirmed the imaging results, we performed immunofluorescence staining on rat brain slices using ATF3 (a marker of cellular stress, also signifies potential neuronal damage) antibody, the results revealed that compared to the sham male group, the SNI male group exhibited a significant increase of ATF3 expression in the CA1. The expression changes of ATF3 in the CA1 region of female rats showed no significant difference between groups (Figure 3E).

### 3.4 Sex differences existed in functional activity of hippocampus region of neuropathic pain rat model

**FIGURE 4:**
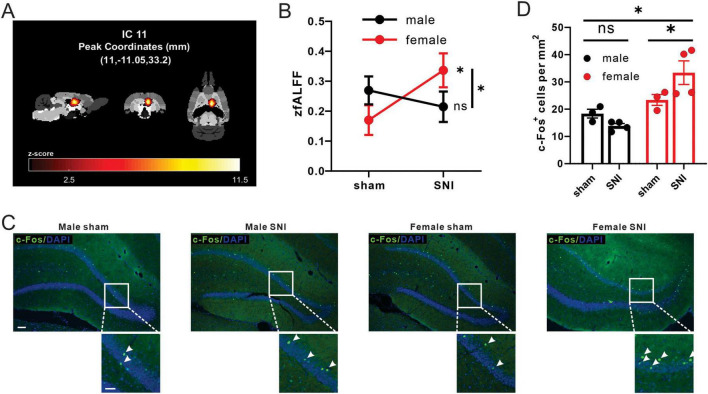
SNI induced differential changes of fALFF values and c-Fos^+^ cell density in male and female rats. **(A)** The front, side, and ventral views of the main area of IC11 in the brain. **(B)** Different alterations of mean zfALFF values of IC11 between SNI groups and sham-operation groups in both male and female (male sham group: *n* = 18; male SNI group: *n* = 18; female sham group: *n* = 20; female SNI group: *n* = 18; * *p* < 0.05, ns: no significance). **(C)** Activation pattern of in DG_R of C-Fos of each group in the DG (male sham-operation group: *n* = 3; male SNI group: *n* = 4; female sham-operation group: *n* = 3; female SNI group: *n* = 4; * *p* < 0.05, ns, no significance; left bar = 100 μm, right bar = 50 μm). **(D)** C-Fos^+^ cell density in DG_R of each group.

Building upon the findings from DTI analysis and indicating the involvement of the microstructural of hippocampus regions in pain-related sex differences, we further analyzed the fMRI data to investigate sex differences in functional activity changes within this region. By calculating the zfALFF value of each IC of the hippocampal region. We found that there were sex differences in the changes of zfALFF values of IC11 (sex × operation interaction: *F* = 4.661, *P* = 0.034). Meanwhile, zfALFF value was significantly increased in the SNI female group compared with the sham female group, but no significant difference was found in male groups (simple effect of operation in males: *F* = 0.556, *P* = 0.458; simple effect of operation in females: *F* = 5.419, *P* = 0.023) (Figures 4A, B).

Furthermore, we validated the neural activity by immunofluorescence staining in dentate gyrus_R (DG_R), the main region in IC11 (Table 1) of neuropathic pain rat model. The interaction effect of c-Fos^+^ cells density between sex and operation was found in DG_R (sex × operation interaction: *F* = 6.590, *P* = 0.028). C-Fos expression was significantly increased in the SNI female group compared with the sham female group, but no significant difference in male groups (simple effect of operation in males: *F* = 1.269, *P* = 0.286; simple effect of operation in females: *F* = 6.269, *P* = 0.031) (Figures 4C, D).

**TABLE 1:** The main regions of each IC in hippocampus region.

Independent Components	Main Region (The number in parenthese represents the number of voxels occupied by the brain region)	Abbreviation	Peak MNI Coordinate (mm)			Peak intensity
			x	y	z	
IC3	Lateral Entorhinal Cortex_L(220)	LEnt_L	−52	−68.05	−2.8	10.5
	Lateral Entorhinal Cortex Internal part_L(145)	ILEnt_L				
	Dentate Gyrus_L(122)	DG_L				
IC5	Lateral Entorhinal Cortex_R(207)	LEnt_R	53	−65.05	0.2	9.8
	Dentate Gyrus R(196)	DG_R				
	Lateral Entorhinal Cortex Internal part_R(129)	ILEnt_R				
IC11	Dentate Gyrus_R(180)	DG_R	11	−11.05	33.2	11.5
	Striatum_R (129)	St_R				
	Fimbria of the Hippocampus2_R(73)	FbHipp2_R				
IC20	Dentate Gyrus_L(223)	DG_L	−7	−35.05	36.2	11.5
	Cornu Ammonis 1_L(164)	CA1_L				
	Fasciola Cinereum_L(62)	FC_L				
IC28	Cornu Ammonis 1_R(140)	CA1_R	29	−20.05	39.2	10
	Cornu Ammonis 3_R(115)	CA3_R				
	Dentate Gyrus_R(93)	DG_R				
IC32	Dentate Gyrus_R(299)	DG_R	11	−32.05	33.2	11.4
	Cornu Ammonis 1_R(152)	CA1_R				
	Fasciola Cinereum_R(79)	FC_R				
IC37	Dentate Gyrus_R(152)	DG_R	2	−23.05	33.2	12.7
	Dentate Gyrus_L(104)	DG_L				
	Fasciola Cinereum_R(50)	FC_R				
IC58	Cornu Ammonis 3_L(317)	CA3_L	−52	−50.05	18.2	5.9
	Cornu Ammonis 1_L(161)	CA1_L				
	Dentate Gyrus_L(137)	DG_L				
IC62	Dentate Gyrus_R(129)	DG_R	38	−62.05	21.2	6.8
	Parasubiculum_L(97)	Psb_L				
	Dentate Gyrus_L(87)	DG_L				

### 3.5 Sex differences existed in global metrics and nodal metrics of hippocampus region of neuropathic pain rat model

**FIGURE 5:**
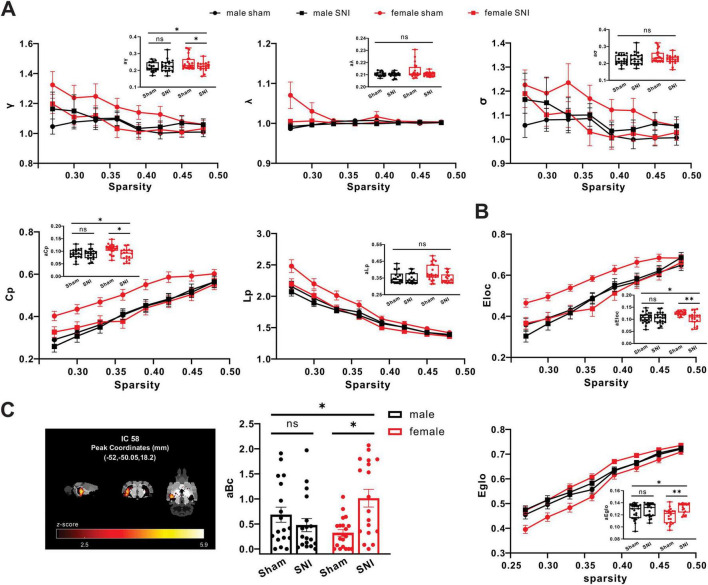
SNI induced changes of small-world network properties in the functional brain networks in rats. **(A)** Different alterations of small-world network properties of hippocampus region between SNI groups and sham groups in both male and female (male sham group: *n* = 18; male SNI group: *n* = 18; female sham group: n = 20; female SNI group: *n* = 18; * *p* < 0.05, ns, no significance). **(B)** Different alterations of parameters of network efficiency of hippocampus region between SNI groups and sham groups in both male and female (male sham group: *n* = 18; male SNI group: *n* = 18; female sham group: *n* = 20; female SNI group: *n* = 18; * *p* < 0.05, ** *p* < 0.01, ns: no significance). **(C)** Different alterations of betweenness centrality of IC58 in hippocampus region between SNI groups and sham groups in both male and female (male sham group: n = 18; male SNI group: *n* = 18; female sham group: *n* = 20; female SNI group: *n* = 18; * *p* < 0.05, ns, no significance).

Building upon the findings from structural and functional analysis and indicating the involvement of the microstructural and activity of hippocampus regions in pain-related sex differences, we further analyzed global and nodal metrics from graph theory to measure the fMRI-network level of male and female rats before and after neural injury. The correlation of mean functional network of hippocampus region (included all ICs) in each group was shown in Supplementary Figure 2. Specifically, in the given threshold range (sparsity range 0.27 - 0.48, 8 grades in total), all subjects exhibited a typical small-world network attribute (γ > 1, λ≈ 1, σ > 1). There were interaction effects between sex and operation of the areas under curves of γ (aγ) and clustering coefficient (aCp) (sex × operation interaction of aγ: *F* = 4.145, *P* = 0.046; sex × operation interaction of aCp: *F* = 4.570, *P* = 0.036). Meanwhile, aγ and aCp were significantly decreased in the SNI female group compared with the sham female group, but no significant difference in male groups (simple effect of operation in males, aγ: *F* = 0.477, *P* = 0.492, aCp: *F* = 0.099, *P* = 0.754, simple effect of operation in females, aγ: *F* = 4.968, *P* = 0.029, aCp: *F* = 11.807, *P* = 0.001) (Figure 5A). In addition, sex differences existed in the variations of the area under curve of local efficiency (aEloc) and global efficiency (aEglo) of hippocampus region (sex × operation interaction of aEg: *F* = 3.985, *P* = 0.049; sex × operation interaction of aEloc: *F* = 5.863, *P* = 0.018). Meanwhile, aEloc was significantly decreased in the SNI female group compared with sham female group, but no significant difference in male groups (simple effect of operation in males: *F* < 0.001, *P* = 0.994; simple effect of operation in females: *F* = 12.084, *P* = 0.001), but aEglo was significantly increased in the SNI female group compared with the sham female group, but no significant difference in male groups (simple effect of operation in males: *F* = 0.596, *P* = 0.443; simple effect of operation in females: *F* = 13.337, *P* < 0.001) (Figure 5B). For nodal metrics, sex differences existed in the variations of the area under curve of betweenness centrality (aBc) of IC58 in hippocampus region (sex × operation interaction: *F* = 11.211, *P* = 0.001). Meanwhile, aBc was significantly increased in the SNI female group (simple effect of operation: *F* = 13.578, *P* < 0.001), but no significant difference in male groups (simple effect of operation: *F* = 1.175, *P* = 0.282) (Figure 5C).

## 4 Discussion

Numerous studies have demonstrated that chronic pain is associated with molecular expression patterns and functional restructuring in the brain (Kuner and Flor, 2017; Mansour et al., 2014). Clinical research has found significant sex differences in the prevalence of chronic pain in humans (Fillingim, 2017). Meanwhile, there is an increasing focus on understanding the pain mechanisms related to sex dimorphism in preclinical research (Delay et al., 2021; Kim and Kim, 2020). Surprisingly, there is scarce research on sex differences in brain neuroplasticity associated with chronic pain. Our study primarily utilizes fMRI technology to investigate the influence of sex in neuroplasticity and adaptive changes in the brain following nerve injury-induced pain hypersensitivity, aiming to fill this research gap in the field. SNI is an animal model of persistent peripheral neuropathic pain (Decosterd and Woolf, 2000). Peripheral neuropathic pain is caused by injury or disease of the somatosensory nervous system, which accounts for 15% - 25% of chronic pain (Fitzcharles et al., 2021; Finnerup et al., 2016). SNI has been broadly used as chronic pain rodent model (Wang et al., 2021; Zhao et al., 2017; Zhang et al., 2021). Studies have confirmed that on day 14 after SNI surgery, the mechanical hypersensitivity of the lateral plantar paw reached the peak period and maintained for a long period, which can simulate the chronic pain state of humans to some extent (Decosterd and Woolf, 2000). Therefore, we conducted fMRI test on day 14 after SNI surgery mainly to reflect the sex differences on the maintaining period of chronic pain. Our results showed that compared to the sham group, both male and female rats exhibited significantly reduced mechanical withdrawal threshold following nerve injury and this reduction persisted until day 14 (experimental endpoint). During this process, there were no significant differences observed in the extent and timing of the withdrawal threshold reduction between the two sexes of rats. Subsequently, we collected and analyzed the signals from fMRI and DTI, utilizing ICA to divide the brain into 64 ICs and assign them to 16 systems. Detailed computational results revealed significant sex dimorphism in both the microstructure and functional activity of the hippocampal system in the rat brain following nerve injury, concurrent with the onset of mechanical pain. In male rats, the microstructure of the hippocampal CA1/CA2 region was significantly compromised, as evidenced by increased MD, RD, and AD values and enhanced ATF3 expression. On the other hand, in female rats, the fALFF value in the hippocampal DG was significantly increased, along with a significant elevation in c-Fos signal. Additionally, the hippocampal system’s small-world network, based on graph theory analysis, also demonstrated marked differences in female rats, primarily characterized by reduced small-world attributes and increased inter-nodal transmission efficiency.

Despite exhibiting similar forms and degrees of chronic pain, the underlying neural mechanisms causing pain in different sexes can exhibit both commonalities and substantial differences, giving the significance of sex differences in chronic pain neural circuits for the development of personalized pain management strategies, including the use of neuromodulation, cognitive therapies, and brain-targeted treatments. Based on previous theories and experimental observations, the persistent and intense nature of chronic pain relies heavily on the state of the limbic system, consisting of the hippocampus and amygdala. The limbic system is a key brain region involved in the nociceptive processing, cognition, and emotion. Sex differences in the amygdala are proposed to underly sex-specific susceptibility to sensory and mood disorders (Seney and Sibille, 2014; Padgaonkar et al., 2020; Andreano et al., 2014). The hippocampal region of interest in this study is a highly plastic structure crucial for processing higher-order information (Leuner and Gould, 2010). Its sex differences involved in anatomical structure and developmental processes have long been recognized (Yagi and Galea, 2019; Yagi et al., 2020). The role of the hippocampus in regulating sensation and emotions also has gained research attention (Deadwyler et al., 1987; Zhu et al., 2019; Su et al., 2023), and we have integrated these aspects using fMRI technology to investigate the importance of hippocampal mediation of sex differences in chronic pain. The hippocampus is well-known to be located in the medial temporal lobe (in cross-section/coronal view), near the temporal horn of the lateral ventricle (coronal view), at the edge of the ambient cistern (in cross-section), and below the amygdala (coronal view). Its structure comprises the hippocampus proper (Ammon’s horn), dentate gyrus (DG), and subiculum, with the hippocampus proper further subdivided into CA1, CA2, CA3, and CA4. Synaptic structures extend from CA4 to CA1, with CA3 also receiving projections from the DG (Fogwe et al., 2024). Our DTI analysis revealed microstructural damage in the CA1/CA2 regions of male SNI rats, along with enhanced expression of the stress signal marker, ATF3. Through literature research, we have found that androgens can promote neuronal repair and axon growth in the central nervous system (Fargo et al., 2008; Bianchi et al., 2020). Additionally, studies have suggested that androgen receptor expression is higher in the CA1 region compared to females (Feng et al., 2010). Given that our data were collected at a time when pain threshold reached its peak and was sustained (14 day after SNI), we speculate that the changes in the CA1/CA2 regions might be related to pain-induced repair mechanisms in males; however, further experimental evidence is needed to support this. Additionally, it is worth exploring the potential connection between the microstructural alterations in CA1/CA2 and susceptibility to androgens. Our results in female rats showed an elevation in fALFF values (indicating neuronal activity) in the DG region following SNI, a phenomenon further confirmed by c-Fos expression. Literature research revealed that there are no significant sex differences in the expression levels of sex hormone receptors in the DG region, but rather morphological differences in granule neuron exist between sexes (Yagi and Galea, 2019). Additionally, the long-term potentiation at the perforant path-dentate gyrus (PP-DG) synapse in the hippocampus of female animals under physiological conditions is smaller compared to male animals (Safari et al., 2021). Therefore, we boldly speculate that the biological mechanisms influencing synaptic plasticity may be the cause of enhanced DG activity in female rats after SNI, implicating their involvement in the maintenance of pain in female animals. Furthermore, graph theory analysis revealed that in female rats, the entire hippocampal region exhibited a reduction in small-world properties and abnormal intrinsic nodal efficiency after pain induction. This finding is in complete alignment with observations from clinical studies (Liu et al., 2011), suggesting that pathological pain may have additional effects on the female hippocampus, potentially leading to disruptions in its microstructure and molecular expression patterns. This necessitates more focused efforts in the future, utilizing higher-resolution techniques for further exploration.

Our study reveals sex differences in the structural and functional activity of the hippocampal system in rats under chronic pain conditions. These changes highlight the intricate interplay between pain processing, neural activity, and hippocampal structural alterations. The findings of altered small-world properties align with clinical observations in human populations, suggesting that results from rodent studies closely mirror clinical phenomena at a more macro level. However, to effectively translate basic research findings into clinical applications, it is essential to include more informative BOLD and DTI data analyses as well as biological validations. The observed microstructure damage in the CA1/CA2 regions of male rats, coupled with the preference of androgen receptor expression in this area (Feng et al., 2010), may provide a theoretical basis for potential treatment strategies targeting precise androgen release in the treatment of chronic pain in males. On the other hand, the overactivity in the DG region and disruption of small-world properties in female rats suggest that interventions targeting the DG region could serve as potential targets for treating chronic pain in females. Importantly, our study underscores the need for heightened attention to sex differences in the structural, functional, and mechanistic aspects of chronic pain. Personalized precision management is vital in clinical practice for the tailored treatment of pain in both sexes, even for the same causal factors.

In summary, our study integrating fMRI and DTI data, reveals sex-specific characteristics of brain functional activity changes associated with chronic pain at the preclinical research level. The results strongly suggested that the hippocampus region played a crucial role in the different mechanisms of chronic pain experienced by male and female. It provides strong support for further exploring the differences of precise chronic pain trigger mechanisms between male and female.

## 5 Limitations

One limitation of this study is that we utilized a rat model for preclinical research. In future studies, further research should be conducted on primates or humans to confirm the generalizability of our findings across species.

## Data Availability

The raw data supporting the conclusions of this article will be made available by the authors, without undue reservation.

## Footnotes

1
http://www.fmrib.ox.ac.uk/fsl/

2
http://www.fil.ion.ucl.ac.uk/spm/

3
https://github.com/trendscenter/gift

4
https://www.alivelearn.net/xjview/

5
http://rfmri.org/dpabi

6
https://www.nitrc.org/projects/gretna/
