# Supplementary material for: Sex differences in functional and structural alterations of hippocampus region in chronic pain: a DTI and resting-state fMRI study
Source: Front Neurosci. 2024 Sep 6;18:1428666. doi: 10.3389/fnins.2024.1428666 (PMC11412943; doi:10.3389/fnins.2024.1428666)
Supplement: Supplementary file 1 [file Data_Sheet_1.docx]

**
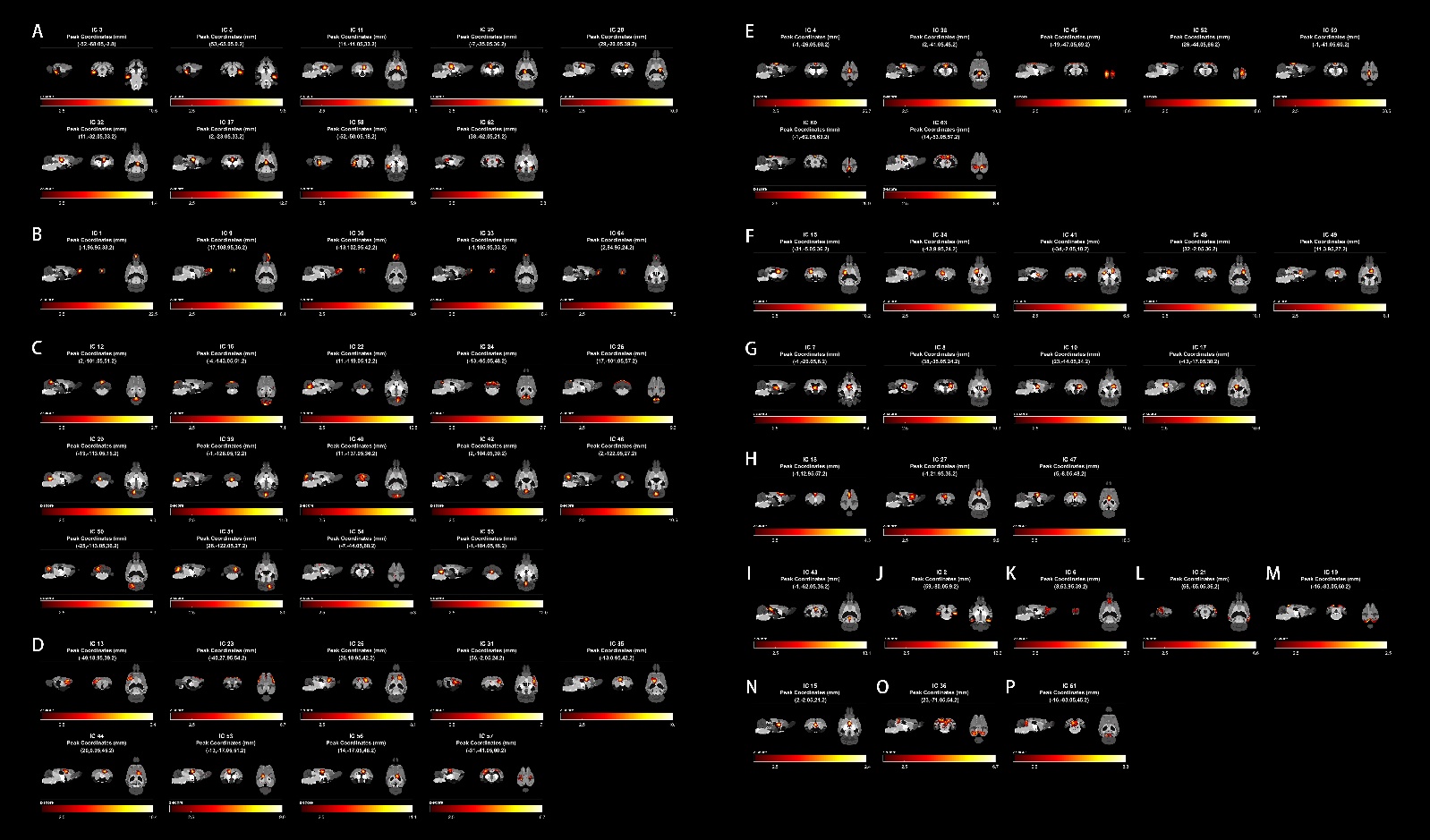
**

**Supplementary figure 1. 64 ICs and 16 systems illustrations of rat brain as calculated by ICA.**

**
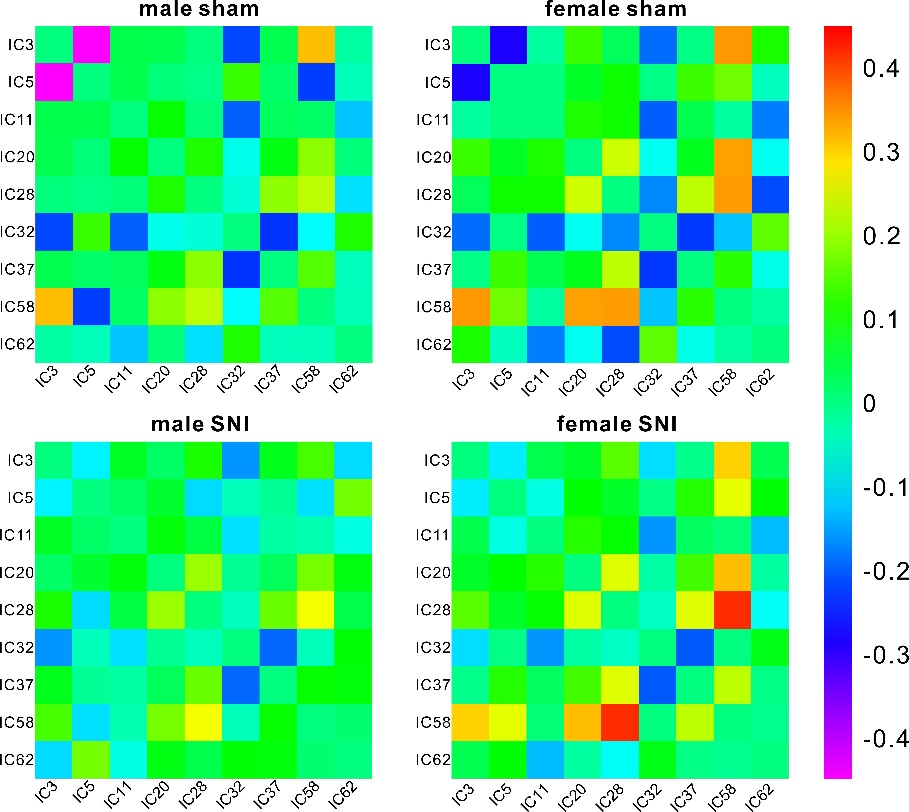
**

**Supplementary figure 2. The correlation of mean functional network of each ICs of hippocampus region.**

**Supplementary Table 1. Main Regions of 64 Independent Components involved in 16 Systems.**

| **Independent Components** | **Main Region** | **Abbreviation** | **MNI Coordinate(mm)** | | | **Peak intensity** |
| --- | --- | --- | --- | --- | --- | --- |
|  |  |  | **x** | **y** | **z** |  |
| Hippocampus Region | | | | | | |
| IC3 | Lateral Entorhinal Cortex_L(220) | LEnt_L | -52 | -68.05 | -2.8 | 10.5 |
| IC5 | Lateral Entorhinal Cortex_R(207) | LEnt_R | 53 | -65.05 | 0.2 | 9.8 |
| IC11 | Dentate Gyrus_R(180) | DG_R | 11 | -11.05 | 33.2 | 11.5 |
| IC20 | Dentate Gyrus_L(223) | DAG_L | -7 | -35.05 | 36.2 | 11.5 |
| IC28 | Cornu Ammonis 1_R(140) | CA1_R | 29 | -20.05 | 39.2 | 10 |
| IC32 | Dentate Gyrus_R(299) | DG_R | 11 | -32.05 | 33.2 | 11.4 |
| IC37 | Dentate Gyrus_R(152) | DG_R | 2 | -23.05 | 33.2 | 12.7 |
| IC58 | Cornu Ammonis 3_L(317) | CA3_L | -52 | -50.05 | 18.2 | 5.9 |
| IC62 | Dentate Gyrus_R(129) | DG_R | 38 | -62.05 | 21.2 | 6.8 |
| Olfactory System | | | | | | |
| IC1 | Glomerular Layer of the Olfactory Bulb_L(163) | GrO_L | -1 | 96.95 | 33.2 | 22.5 |
| IC9 | Olfactory Bulb_R(598) | OB_R | 17 | 108.95 | 36.2 | 8.4 |
| IC30 | Olfactory Bulb_L(929) | OCB_L | -13 | 102.95 | 42.2 | 8.9 |
| IC33 | Olfactory Bulb_L(242) | OCB_L | -1 | 105.95 | 33.2 | 10.4 |
| IC64 | Glomerular Layer of the Olfactory Bulb_R(174) | GrO_R | 2 | 84.95 | 24.2 | 7.9 |
| Cerebellum | | | | | | |
| IC12 | Molecular Layer of the Cerebellum_R(456) | MoCb_R | 2 | -101.05 | 51.2 | 12.7 |
| IC16 | Molecular Layer of the Cerebellum_L(595) | MCC_L | -4 | -143.05 | 51.2 | 7.1 |
| IC22 | Granule Cell Layer of the Cerebellum_R(444) | GrCb_R | 11 | -119.05 | 12.2 | 10.5 |
| IC24 | Molecular Layer of the Cerebellum_L(641) | MCC_L | -13 | -95.05 | 48.2 | 7.7 |
| IC26 | Molecular Layer of the Cerebellum_R(519) | MoCb_R | 17 | -101.05 | 57.2 | 9.3 |
| IC29 | Granule Cell Layer of the Cerebellum_L(471) | GUC_L | -13 | -113.05 | 15.2 | 9 |
| IC39 | Molecular Layer of the Cerebellum_L(433) | MCC_L | -1 | -128.05 | 12.2 | 11 |
| IC40 | Molecular Layer of the Cerebellum_R(883) | MoCb_R | 11 | -137.05 | 36.2 | 6.8 |
| IC42 | Molecular Layer of the Cerebellum_R(400) | MoCb_R | 2 | -104.05 | 30.2 | 12.4 |
| IC48 | Molecular Layer of the Cerebellum_R(481) | MoCb_R | 2 | -122.05 | 27.2 | 10.5 |
| IC50 | Molecular Layer of the Cerebellum_L(1312) | MCC_L | -25 | -113.05 | 36.2 | 6.9 |
| IC51 | Molecular Layer of the Cerebellum_R(1237) | MoCe_R | 26 | -122.05 | 27.2 | 8 |
| IC54 | Molecular Layer of the Cerebellum_R(221) | MCC_R | -7 | -44.05 | 60.2 | 5.3 |
| IC55 | Molecular Layer of the Cerebellum_L(321) | MCC_L | -1 | -104.05 | 18.2 | 12 |
| Periaqueductal Gray | | | | | | |
| IC43 | Periaqueductal Gray_R(207) | PAG_R | -1 | -62.05 | 36.2 | 13.1 |
| Perihinal Area | | | | | | |
| IC2 | Perirhinal Area 36_R(161) | PRh36_R | 59 | -80.05 | 9.2 | 12.6 |
| Prelimbic System | | | | | | |
| IC6 | Prelimbic Cortex_R(348) | PrL_R | 8 | 63.95 | 39.2 | 6.7 |
| Auditory System | | | | | | |
| IC21 | Primary Auditory Cortex_R(433) | A1_R | 68 | -65.05 | 36.2 | 6.6 |
| Primary Cingular cortex | | | | | | |
| IC18 | Primary Cingular Cortex_L(314) | PCC_L | -1 | 12.95 | 57.2 | 14.3 |
| IC27 | Primary Cingular Cortex_L(313) | PCC_L | -1 | 21.95 | 36.2 | 9.9 |
| IC47 | Primary Cingular Cortex_R(196) | PCC_R | 5 | -8.05 | 48.2 | 10.3 |
| Sensorimotor System | | | | | | |
| IC13 | Primary Somatosensory Cortex Upperlips_L(368) | S1ULp_L | -49 | 18.95 | 39.2 | 6.4 |
| IC23 | Primary Motor Cortex_L(213) | M1_L | -40 | 27.95 | 54.2 | 6.7 |
| IC25 | Primary Motor Cortex_R(287) | M1_R | 26 | 18.95 | 42.2 | 8.1 |
| IC31 | Primary Somatosensory Cortex Barrel Field_R(466) | S1BF_R | 56 | -2.05 | 24.2 | 7.1 |
| IC35 | Primary Motor Cortex_L(222) | M1_L | -13 | 0.95 | 42.2 | 10.1 |
| (Continued) | | | | | | |
| IC44 | Primary Motor Cortex_R(265) | M1_R | 20 | 0.95 | 45.2 | 10.4 |
| IC53 | Primary Motor Cortex_L(139) | M1_L | -13 | -17.05 | 51.2 | 9 |
| IC56 | Primary Moter Cortex_R(130) | M1_R | 14 | -17.05 | 48.2 | 11.1 |
| IC57 | Primary Somatosensory Cortex Barrel Field_L(204) | S1BF_L | -31 | -41.05 | 60.2 | 6.7 |
| Visual System | | | | | | |
| IC19 | Primary Visual Cortex Monocular Area_L(154) | V1M_L | -16 | -83.05 | 60.2 | 12.5 |
| Retrosplenial Cortex | | | | | | |
| IC4 | Retrosplenial Dysgranular Cortex_L(133) | RSD_L | -1 | -26.05 | 60.2 | 26.7 |
| IC38 | Retrosplenial Granular Cortex Part B_R(111) | RSGb_R | 2 | -41.05 | 45.2 | 10.8 |
| IC45 | Retrosplenial Dysgranular Cortex_L(237) | RSD_L | -19 | -47.05 | 69.2 | 10.6 |
| IC52 | Retrosplenial Dysgranular Cortex_R(299) | RSD_R | 26 | -44.05 | 66.2 | 10.8 |
| IC59 | Retrosplenial Dysgranular Cortex_L(264) | RSD_L | -1 | -41.05 | 60.2 | 28.5 |
| IC60 | Retrosplenial Dysgranular Cortex_L(315) | RSD_L | -1 | -62.05 | 63.2 | 26.9 |
| IC63 | Retrosplenial Dysgranular Cortex_R(157) | RSD_R | 14 | -53.05 | 57.2 | 8.4 |
| Septal Region | | | | | | |
| IC15 | Septal Region_R(181) | SR_R | 2 | -2.05 | 21.2 | 12.4 |
| Striatum | | | | | | |
| IC14 | Striatum_L(217) | St_L | -31 | -5.05 | 36.2 | 10.2 |
| IC34 | Striatum_L(559) | St_L | -13 | 9.95 | 24.2 | 8.9 |
| IC41 | Striatum_L(628) | St_L | -34 | -2.05 | 18.2 | 6.5 |
| IC46 | Striatum_R(371) | St_R | 32 | -2.05 | 36.2 | 10.1 |
| IC49 | Striatum_R(192) | St_R | 11 | 3.95 | 27.2 | 8.1 |
| Thalamus | | | | | | |
| IC7 | Thalamus_L(395) | Th_L | -1 | -20.05 | 6.2 | 8.4 |
| IC8 | Thalamus_R(536) | Th_R | 38 | -35.05 | 24.2 | 10.3 |
| IC10 | Thalamus_R(410) | Th_R | 23 | -14.05 | 24.2 | 10 |
| IC17 | Thalamus_L(264) | Th_L | -13 | -17.05 | 30.2 | 10.6 |
| Superior Colliculus | | | | | | |
| IC36 | Deeper Layers of the Superior Colliculus_R(250) | DpSC_R | 23 | -71.05 | 54.2 | 6.7 |
| Inferio Colliculus | | | | | | |
| IC61 | External Cortex of the Inferior Colliculus_L(199) | ECIC_L | -16 | -83.05 | 45.2 | 8.8 |

**Supplementary Table 2. Statistical parameters of DTI analysis.**

| **Brain regions** | **DTI metrics** | **Interaction Effect** | **Simple Effect** | | **Main Effect** | |
| --- | --- | --- | --- | --- | --- | --- |
|  |  |  | **Male** | **Female** | **Operation** | **Sex** |
| CA1_L | MD | *F* = 5.332 | *F* = 6.525 | *F* = 0.552 |  |  |
|  |  | *P* = 0.023^*^ | *P* = 0.013^*^ | *P* = 0.460 |  |  |
|  | AD | *F* = 2.769 |  |  | *F* = 0.243 | *F* = 0.029 |
|  |  | *P* = 0.100 |  |  | *P* = 0.624 | *P* = 0.864 |
|  | RD | *F* = 5.030 | *F* = 7.090 | *F* = 0.298 |  |  |
|  |  | *P* = 0.028^*^ | *P* = 0.009^**^ | *P* = 0.587 |  |  |
|  | FA | *F* = 0.657 |  |  | *F* = 1.875 | *F* = 1.918 |
|  |  | *P* = 0.420 |  |  | *P* = 0.175 | *P* = 0.170 |
| CA1_R | MD | *F* = 4.820 | *F* = 6.165 | *F* = 0.428 |  |  |
|  |  | *P* = 0.031^*^ | *P* = 0.015^*^ | *P* = 0.515 |  |  |
|  | AD | *F* = 2.394 |  |  | *F* = 0.096 | *F* = 0.094 |
|  |  | *P* = 0.126 |  |  | *P* = 0.757 | *P* = 0.760 |
|  | RD | *F* = 4.687 | *F* = 7.251 | *F* = 0.167 |  |  |
|  |  | *P* = 0.033^*^ | *P* = 0.009^**^ | *P* = 0.684 |  |  |
|  | FA | *F* = 1.257 |  |  | *F* = 2.930 | *F* = 0.725 |
|  |  | *P* = 0.266 |  |  | *P* = 0.091 | *P* = 0.397 |
| CA2_L | MD | *F* = 4.620 | *F* = 3.795 | *F* = 1.255 |  |  |
|  |  | *P* = 0.035^*^ | *P* = 0.055 | *P* = 0.272 |  |  |
|  | AD | *F* = 2.328 |  |  | *F* = 1.874 | *F* = 0.070 |
|  |  | *P* = 0.131 |  |  | *P* = 0.175 | *P* = 0.793 |
|  | RD | *F* = 4.492 | *F* = 7.942 | *F* = 0.050 |  |  |
|  |  | *P* = 0.037^*^ | *P* = 0.006^**^ | *P* = 0.823 |  |  |
|  | FA | *F* = 0.512 |  |  | *F* = 9.052 | *F* = 0.875 |
|  |  | *P* = 0.476 |  |  | *P* = 0.003^**^ | *P* = 0.352 |
| CA2_R | MD | *F* = 6.161 | *F* = 5.768 | *F* = 1.280 |  |  |
|  |  | *P* = 0.015^*^ | *P* = 0.019^*^ | *P* = 0.261 |  |  |
|  | AD | *F* = 4.584 | *F* = 0.711 | *F* = 4.676 |  |  |
|  |  | *P* = 0.035^*^ | *P* = 0.402 | *P* = 0.034 |  |  |
|  | RD | *F* = 4.437 | *F* = 8.902 | *F* = 0.002 |  |  |
|  |  | *P* = 0.038^*^ | *P* = 0.004 | *P* = 0.963 |  |  |
|  | FA | *F* = 0.014 |  |  | *F* = 7.931 | *F* = 0.002 |
|  |  | *P* = 0.905 |  |  | *P* = 0.006^**^ | *P* = 0.965 |

*CA1_L: Cornu Ammonis1_L; CA1_R: Cornu Ammonis1_R; CA2_L: Cornu Ammonis2_L; CA2_R: Cornu Ammonis2_R.*

** P <0.05; ** P < 0.*
